# Novel polymer coating for chemically absorbing CO_2_ for safe Li-ion battery

**DOI:** 10.1038/s41598-020-67123-1

**Published:** 2020-06-25

**Authors:** Jean-Christophe Daigle, Yuichiro Asakawa, Alexis Perea, Martin Dontigny, Karim Zaghib

**Affiliations:** 1 Center of Excellence in Transportation Electrification and Energy Storage (CETEES), Hydro-Québec, 1806, Lionel-Boulet Blvd., Varennes, Quebec, J3X 1S1 Canada; 2 Murata Munufacturing, 10-1 Higashikotari 1-chrome, Nagaokakyo-shi, Kyoto, 617-8555 Japan

**Keywords:** Materials science, Materials for energy and catalysis, Batteries

## Abstract

Gas evolution in Li-ion batteries remains a barrier for the implementation of high voltage materials in a pouch cell format; the inflation of the pouch cell is a safety issue that can cause battery failure. In particular, for manganese-based materials employed for fabricating cathodes, the dissolution of Mn^2+^ in the electrolyte can accelerate cell degradation, and subsequently gas evolution, of which carbon dioxide (CO_2_) is a major component. We report on the utilization of a mixture of polymers that can chemically absorb the CO_2_, including the coating of aluminum foils, which serve as trapping sheets, introduced into two Ah pouch cells—based on a LiMnFePO_4_ (cathode) and a Li_4_Ti_5_O_12_ (anode). The pouch cells with trapping sheets experienced only an 8.0 vol% inflation (2.7 mmol CO_2_ per gram of polymers) as opposed to the 40 vol% inflation for the reference sample. Moreover, the cells were cycled for 570 cycles at 1 C and 45 °C before reaching 80% of their retention capacity.

## Introduction

The continuous quest for an inexpensive, high-energy battery for electrical vehicles (EVs) and energy storage (ES) applications has pushed industrial scientists to develop cells with high energy density by weight and volume. Optimizing the capacity of active materials in electrodes, and stacking increasingly more electrodes into a large format cell has become a valid path to increasing the energy density in battery packs, and minimizing their cost^1–4^. On the other hand, safety should not be compromised, which is a real concern for consumers as no one wants to travel on potential “rolling bombs.”

The implementation of a cathode with a high capacity and voltage to increase the battery’s energy density is in vogue; consequently, manganese (Mn) or cobalt-based (Co) cathode materials have become prevalent in the production of high energy batteries. However, since the cost of Mn is lower than that of Co, reducing Co in cathode materials is a common approach to reduce cell costs^5^. However, increasing the Mn content in active materials can cause other problems. The unwanted dissolution of Mn^2+^ in the electrolyte (also initiated by water and HF) during cell operations can cause myriad side effects such as deposits on the anode surface and a reaction with the electrolyte where the liquid electrolyte may degrade rapidly during the first few cycles to produce gas. Gas evolution during cycling is one of the major problems associated with (especially) Mn-based cathodes. These gases principally constitute CO_2_, which originates from the decomposition of the liquid electrolyte in the presence of Mn ions^6–8^. In the case of LMO, reaction occurs on the surface with Li^+^ and Mn^2+^, and they are leached from the surface, therefore there is more Li ions migrate from the core, consequently electrons hopping from Mn^3+^ to Mn^4+^. The disproportionate reaction of Mn^3+^ generates Mn^2+^/Mn^4+^ which diffuses in electrolytes; studies with LMFP are limited. The degradation of the solvents in the presence of a Li_4_Ti_5_O_12_ (LTO) anode and initiated by PF_5_^-^ has also been reported as a source of CO_2_ and other gases^9,10^. Moreover, a paper published in 2019 reported that CO_2_ was found during the thermal runaway of all types of Li-ion batteries^8^. This problem remains a more substantial one for Mn-based materials (LiMn_2_O_4_ (LMO), LiMn_x_Fe_1-x_PO_4_ (LMFP), LiNiMnCoO_2_ (NMC), etc.). This is a serious concern for the operation of pouch cell format batteries because battery cell expansion can be a safety issue^11^.

Many strategies have been documented in order to reduce the amount of Mn^2+^ dissolved in the electrolyte. Frequently, molecules with ionic or Van der-Waals interactions with bivalent ions have been used to trap ions and prevent the decomposition of electrolytes. For example, aza crown and crown ether derivative polymers have been used as separators in Li-ion batteries for trapping M^2+^ with some success, significantly improving the cycle life of cells^12–15^. Crown-ether derivatives have been very efficient for trapping; the grafting on of carbon (acetylene black) was demonstrated to be efficient for capturing Mn^2+^, and extended the cycle life of a NMC-graphite Li-ion cell^14^. A. Banerjee *et al*. introduced a multifunctional separator based on a poly(ethylene-alt-maleic anhydride) commercial polymer, to capture Mn^2+^ by chelation with –COOH groups^16^. The amount of Mn^2+^ detected on the negative electrode was 1.5 times lower than that of plain separators, and a higher capacity was retained by cells (LMO-graphite) at 30 °C and 55 °C. A novel binder for fabricating cathodes was also investigated, with H. Lee *et al*. reporting an alginate-based polymer being used as a binder for LMO-based batteries^17^. Dissolving Mn^2+^ in the electrolyte was reduced drastically compared with a PVDF binder at elevated temperatures, the chemical coordination between the secondary alcohol groups from the binder and Mn^2+^ impeding the diffusion of ions in the electrolyte.

Chemical modification of separators, binders or carbons are elegant methods to produce functional materials efficient for trapping ions. However, the application of new materials in commercial products is difficult because integration into battery production lines is so costly.

Instead of using new materials for the fabrication of a cathode or battery, we propose, for the first time, the use of active polymers to capture the CO_2_ produced during cycles^6,18,19^, to chemically prevent pouch cell inflation instead of mechanically controlled. Combining two polymers and one catalyst in the solid phase offers a good system for trapping CO_2_^20–23^. This system can be used as a polymeric coating on aluminum foil introduced as a layer in a pouch cell. This approach is unique and does not require any modification of the production line, offering a cost-effective approach for battery manufacturers. We introduced this concept in two Ah pouch cells composed of a stack of cathodes of carbon-coated LiMnFePO_4_ (LMFP), anodes of carbon-coated Li_4_Ti_5_O_12_ (LTO) and a liquid electrolyte.

## Results and Discussion

**Figure 1:**
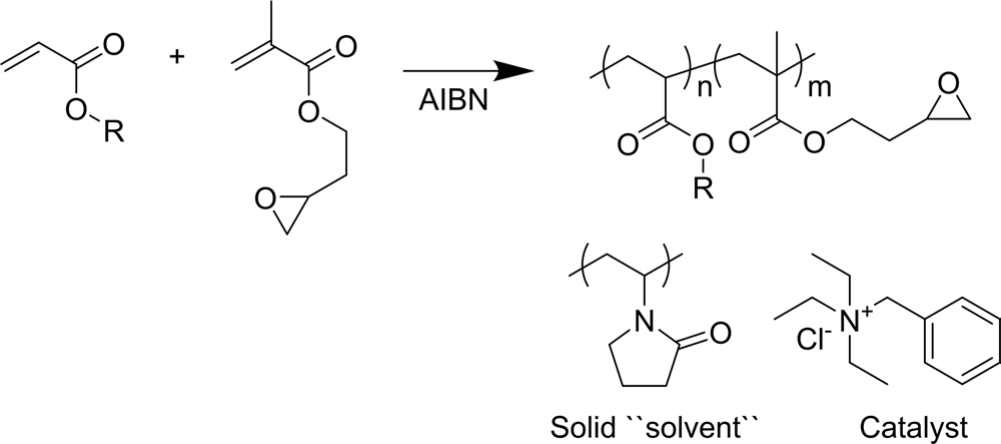
Mixture used as a coating to capture CO_2_.

S.-I. Yamamoto *et al*., reported in 2005 a polymer mixture which can capture gaseous CO_2_ under atmospheric pressure^21^. This system was unique as the polymers were applied in a solid form with glycidyl-methacrylate-based polymers (active polymer), poly(N-vinyl-2-pyrrolidone) (PVP) as a solid solvent and LiBr as a catalyst. The epoxy groups reacted with CO_2_ to form cyclic carbonate and a conversation of 94% was reported. PVP was applied as a good phase transfer agent, acting as a “solid solvent” for the catalyst and permitting a mixture with glycidyl based polymers. To our knowledge, that was the only example of trapping CO_2_ with polymers in the solid phase. We decided to use a more efficient and polymer-soluble catalyst in our system^22^, which is highlighted in Fig. 1.

**Figure 2:**
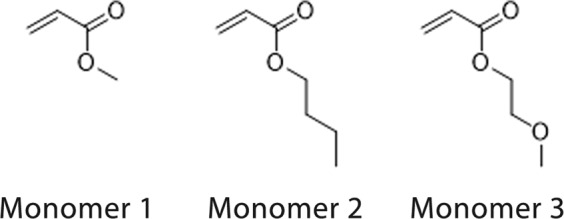
Monomers copolymerized with glycidyl methacrylate and tested in this study.

Because of the nature of the process, the injection of electrolytes into cells wet all parts, and as such the coating has to meet several requirements: it should be insoluble in solvents, it should capture gas, and it should exhibit excellent adhesion to aluminum (Al) foil. The polymers were modified by incorporation of monomers into the backbone in order to increase adhesion to Al and to evaluate effect on gas capture. Three (3) monomers with glycidyl methacrylate were tested as copolymers in order to evaluate the adhesion to the Al foil and the effect of CO_2_ capture. Figure 2 shows these monomers. Examples of the ^1^H NMR spectra (Figure S1-S3) and GPC traces (Figure S4-S5) can be found in the Supplementary Information.

**Figure 3:**
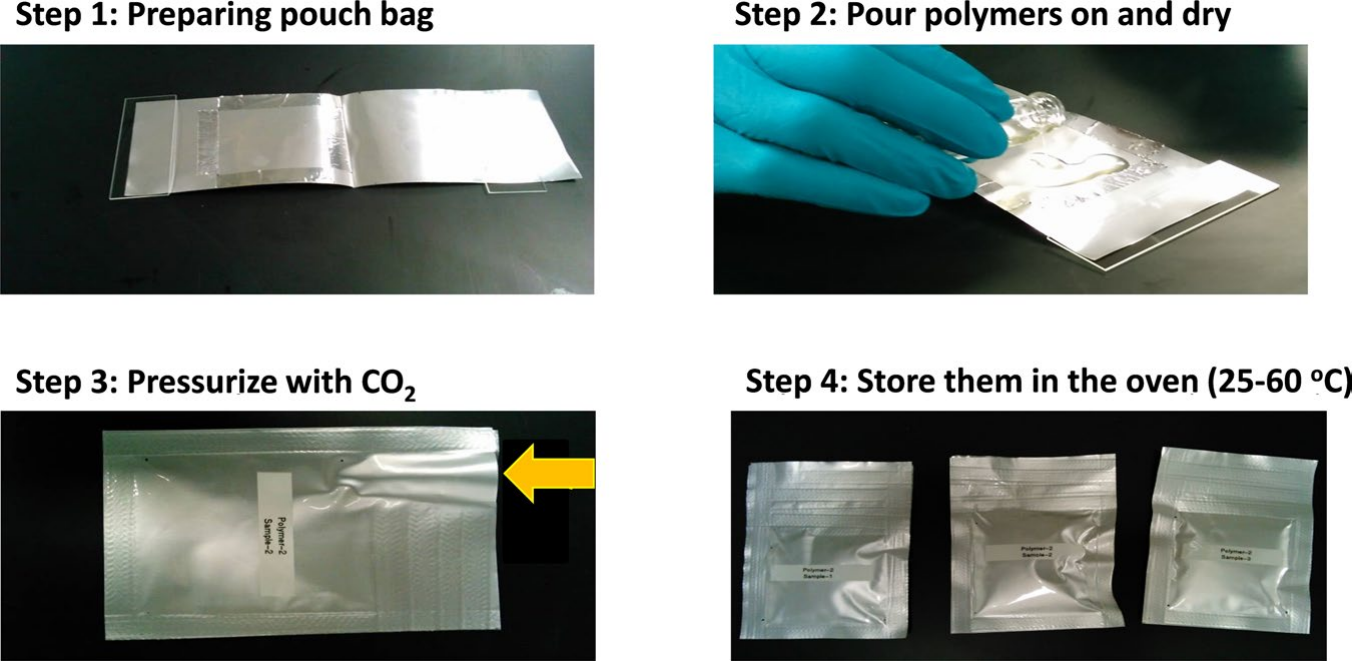
Steps for testing CO_2_ trapping by polymers.

The polymers were mixed using the following method. First, a solid solvent (PVP) and catalyst were mixed with ethanol until complete dissolution (0.15 g, 50.0 mg (5.0 mol% versus copolymer) and 1.00 g., respectively). A second solution of copolymer (0.50 g. and 1.5–2.0 g of NMP) was heated at 80 °C under vigorous stirring. Drop by drop, the first solution was added to the second under vigorous stirring, the final solution was then cooled at 22 °C to obtain a homogenous mixture. This method ensured the perfect mixed of polymers without phase separation. The initial evaluation of CO_2_ trapping was done using a simply dropping mixture (80.00–100.00 mg) on an Al-plastic bag used for making the pouch cell; the testing for the chemical absorption of gas was done by pressurizing with CO_2_ (5.00–10.00 mg) an Al-plastic bag as shown in Fig. 3. All weights were measured using an electronic balance with an accuracy of 0.01 mg. Sealed Al-plastic bags were left in an oven at 25 °C, 45 °C, and 60 °C for different periods of time. This simple method was selected because a high pressure did not occur in the pouch cell, and cells may well vent at low pressures which presents safety concerns.

**Figure 4:**
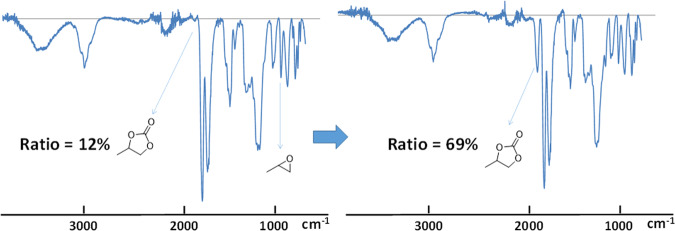
FTIR spectra of poly(glycidyl methacrylate-co-methyl acrylate) (Monomer 1) coating of 30 μm on Al foil after 12 h at 45 °C.

The conversion of the epoxy groups in cyclic carbonate was followed by Fourier transform infrared spectroscopy (FTIR)^21^. The conversion rate was maybe overestimated due to the limited penetration depth by IR. Therefore, we cannot scrutinize the 30 μm tick of coatings, we assumed a depth around 5 μm. The disappearance of signals at 900–905 cm^−1^ (epoxy groups) and the appearance of C=O characteristic of cyclic carbonate at 1790–1800 cm^−1^ allowed an easy calculation of the conversion using the ratio of the two areas (Fig. 4). Solid-state ^13^C NMR was also performed to confirm the presence of cyclic carbonate, and the appearance of C=O groups at 155 ppm (see Figure S6 in Supplementary Information).

**Figure 5:**
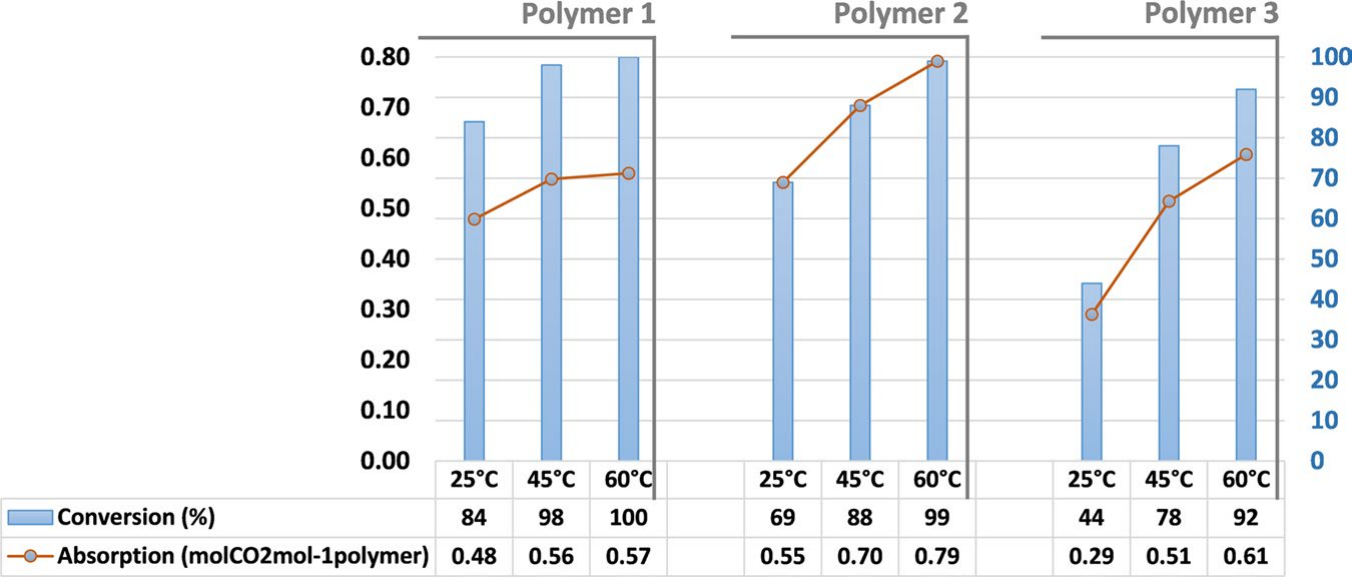
Diagram bars of the conversion of epoxy groups calculated using the area of peaks by FTIR and lines showing chemical absorption calculated using eq. S1.

Figure 5 shows the results of the conversion calculated using data collected by FTIR for the three polymers at different temperatures after 24 h of gas exposure. Chemical absorption of CO_2_ by the polymers was calculated using conversion (area of peaks by FTIR) and the amount of active polymer coated (eq. S1 in Supplementary Information).

The rate of conversion of oxirane moieties with CO_2_ seemed to be correlated with the nature of the monomer. Indeed, the methyl acrylate (monomer 1) based polymer (polymer 1) had a rapid conversion compared to monomer 2 (polymer 2), despite the fact that polymer 2 had a higher level of glycidyl methacrylate incorporated (80 mol% vs 57 mol%, see Table 1). We deem it to be plausible that this was related to the proximity between the polymer chains because the short pendant groups diminished the free volume between the polymers. Therefore, PVP and the solubilized catalyst were closer to poly(glycidyl methacrylate-co-methyl acrylate) (polymer 1) thus oxirane moieties reacted rapidly because of the catalysts in this environment^21^. Monomer 3 based polymer (Polymer 3), with a bulky pendant group (ethylene glycol methyl ether), had the slowest conversion of the three polymers, which corroborated our previous observations. Moreover, the logically augmented levels of trapping groups in the polymer backbones (polymer 2 vs. polymer 1 and 3) permitted the capture of more CO_2_ (polymer 2: 0.79 molCO_2_mol^−1^polymer). The time of exposure was also evaluated; polymer 1 had a conversion of 87% after 12 h at 45 °C and 98% after 24 h. An important fact is that the conversion of the epoxy groups was almost complete at 60 °C for all polymers after 24 h. As reported by Yamamoto *et al*.^21^. NMP is a better solvent for ionic catalyst and PVP. To validate that we changed NMP with tetrahydrofuran, and experienced a conversion decrease of 17% (45 °C, 24 h). Moreover, a decrease in the catalyst quantity in the mixture reduced the conversion of the oxirane groups to 48% with 2.5 mol% from 65% with 5.0 mol%, as expected.

**Table 1:** Experimental details.

Monomer	Amount [g]	n [mmol]	Initiator [mg]	Yield [g]	Mn^a^ [gmol^−1^]	PDI^a^	y^b^ [mol%]
Methyl acrylate (MA)	3.5	40.7	88	6.9	28,900	1.9	57
n-Butyl acrylate (nBA)	2.0	15.6	80	5.8	30,900	1.8	80
Ethylene glycol methyl ether acrylate (EGA)	2.8	21.5	76	6.0	23,700	2.5	66
3-(trimethoxysilyl)propyl methacrylate (TMSPA)	3.0	12.1	89	5.3	21,300	2.0	92

^a^Determined by GPC in THF at 25 °C equipped with triple detection. ^b^GMA incorporated in polymer backbone determined by ^1^H NMR.

Moreover, the physical absorption of CO_2_ was related to the nature of the comonomer, and not with the level of incorporation in the main chains of glycidyl methacrylate alone. Many research teams have reported that the polymer itself physically interacted with the gas; reports of the solubilizing of supercritical CO_2_ by polymers have suggested that the solubility of CO_2_ was driven by the free volume of polymers^24,25^. This behavior can change the nature of the coating, assuming that n-butyl acrylate (Monomer 2) based polymers have a higher free volume compared with methyl acrylate based polymers; free volume being related to the temperature of the glass transition (T_g_). Statistical random copolymers with Monomer 2 had lower T_g_ versus Polymer 1 established on the Fox equation^26^, calculated at 32 °C and 43 °C respectively. Moreover, at low pressures the solubility of the gas increased with pressure with a diluent effect, acting like a plasticizer (increasing the distance between polymer chains), and therefore decreasing T_g_^27^. Therefore, in the presence of CO_2_, the polymer incorporating n-butyl acrylate becomes very fluid and exhibits a lack of adhesion after CO_2_ exposure, which could be related to that along with the hydrophobicity of Monomer 2.

We assumed that the thickness of the coating had an influence on the absorption, and as such we evaluated the coating of polymer 1 using the Doctor Blade method on a large Al foil. We decided to use poly(glycidyl methacrylate-co-methyl acrylate) (polymer 1) due to its better adhesion on Al foil and fast trapping was observed. The solution was coated using the Doctor Blade method on Al Foil and dried at 80 °C for 12 h. The coating (surface = 45 cm^2^, thickness = 81 μm, 123 mg of polymers) was then inserted into an Al-Plastic bag pressurized with CO_2_. We evaluated the coating at 45 °C, the testing temperature of the cells. After 12 h, a conversion of 69% of the epoxy groups was observed by FTIR. However, the coating showed a lack of adhesion when soaked in battery electrolyte (20 mL of electrolyte for 2 h at 22 °C), and the polymers were soluble. Consequently, 3-(trimethoxysilyl)propyl methacrylate was selected as a monomer for polymerization with glycidyl methacrylate. This monomer is known to form a strong bond with aluminum oxide preventing the leaching of the coating from the Al surface^28^. Moreover, we incorporated a large percentage of glycidyl methacrylate (92 mol%) in polymer backbone in order to enhance its chemical absorption. The coating on the Al foil was homogenous and did not exhibit any dissolution in the battery electrolyte (the same proportion of solid solvent and catalyst which were used previously, were mixed).

**Figure 6:**
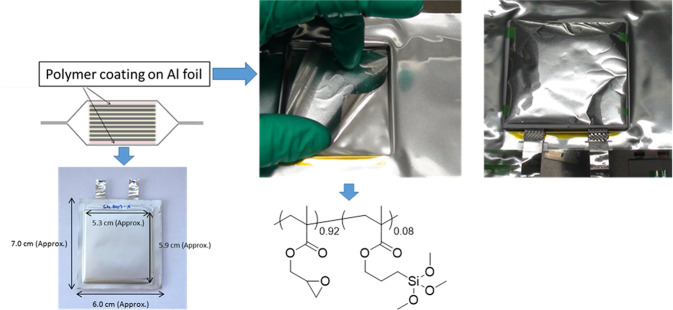
Configuration of two Ah pouch cells with trapping sheets.

The coating on the Al foil is shown in Fig. 6. In brief, two sheets of 27.5 cm^2^ were introduced into the pouch cell, 61.0 mg of polymers by sheet with a thickness of 90 μm. A pouch cell of 2 Ah was assembled with an LMFP (cathode) and LTO (anode), aluminum foils with polymer coatings were added to the top and the bottom of the cell (two sheets) (Fig. 6). Details of the electrode preparations and cell configurations are highlighted in the Methods Section.

**Figure 7:**
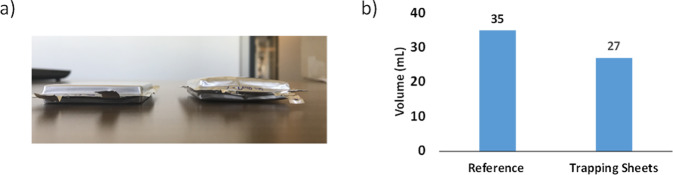
(**a**) Image of the cells with polymer trapping sheets (left) and without trapping sheets (right). (**b**) Diagram bars of the cell volume after 3 cycles at 0.2 C and 25 °C.

Prior to being tested, the cells were cycled at 0.2 C for 3 cycles (charge and discharge) at 25 °C (30 h of cycling), the charge-discharge curves of which can be found in the Supplementary Information (Figure S7). Images of the cells after cycling and their respective diagram bars are shown in Fig. 7. Visually (Fig. 7a), we observed the inflation of the cell without trapping sheets. We expected a major gas evolution during the first cycles as reported in the literature^6,19^. We measured the volume of the cells using Archimedes’ principle, initially reported by J. R. Dahn and team to evaluate the gas evolution in NMC-graphite cells^29^. The initial volumes of the cells prior to being cycled were 25.0 mL, the cell without sheets grew 40 vol% (10.0 mL of gas was generated in the reference) while the cell with trapping sheets grew only 8.0 vol% which was not visually detectable. Based on Fig. 7b, we calculated the amount of gas trapped by the two sheets (i.e. 8.0 mL of gas, which we assumed was CO_2_ since the polymers did not chemically interact with the other gas). Using the ideal gas law (or general gas equation) for our calculations at 1 bar pressure^19^, we estimated that 2.7 mmol of CO_2_ per gram of polymers was trapped.

**Figure 8:**
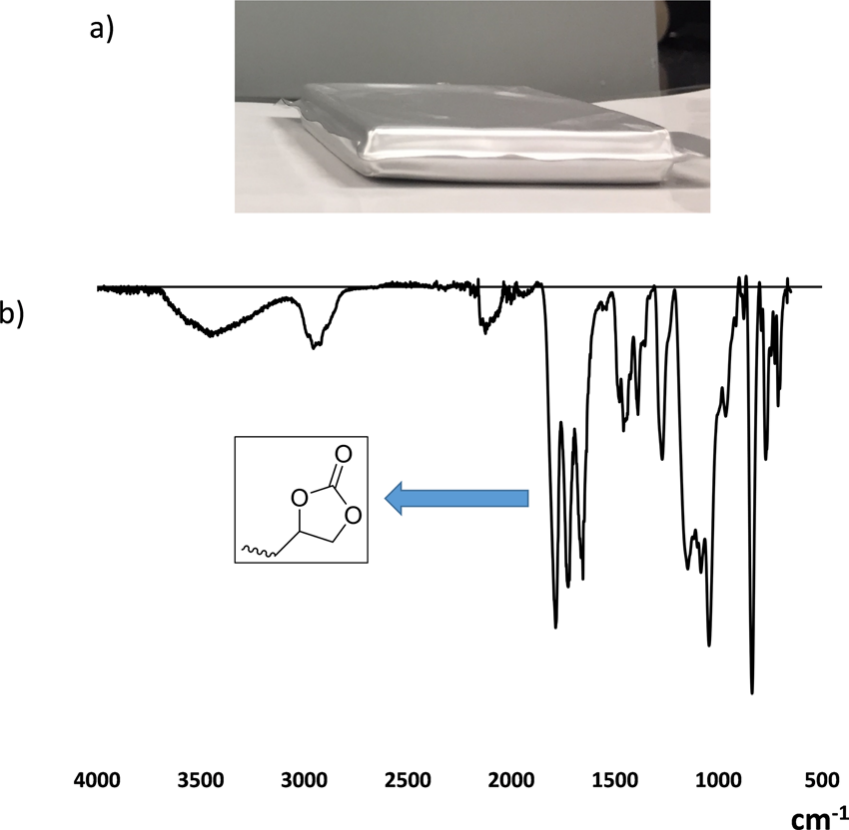
(**a**) Image of cell with trapping sheets after 570 cycles at 1 C and 45 °C. (**b**) FTIR spectrum of the trapping sheet after disassembly of the cycled cell.

The cells were extensively cycled to age the active materials and accelerate the side reactions. Although only a minimum of gas was generated after the first cycle, we deemed it valuable to evaluate the technology over a long cycle time. The cells had a capacity of 1.90 Ah at 1 C and 45 °C (first cycle), and their retention capacity was still at 99% over an extended 290 cycles. A depletion of capacity was recorded up to 1.52 Ah after 570 cycles (retention capacity <80%), (see Figure S8 for the cycle-life curve), and no cell inflation was visually observed while reference had inflation (see Figure S9 in Supplementary Information). The disassembly of the cell allowed the recovery of the trapping sheets, which exhibited no visual degradation or leaching; subsequent analysis by FTIR showed a clean spectrum without trace of degradation, presenting a large peak at approximately 1800 cm^−1^ (Fig. 8).

## Conclusion

We demonstrated, for the first time, the effectiveness of applying a coating of polymers on Al foil for chemically absorbing CO_2_ during Li-ion pouch cell operations. This strategy was effective in preventing pouch inflation. We observed a decrease in the cell volume (after cycling) by 80% as the sheets captured CO_2_. No safety issues were experienced due to cell inflation. Moreover, this technology was applied to a 2 Ah Li-ion battery which was cycled over more than 500 cycles at 1 C and 45 °C in order to accelerate the aging process (with associated side reactions). Therefore, we can conclude that our approach is economically viable, industrially applicable, and permits the fabrication of safe high-power Li-ion batteries based on large format cell with significant cycle life potential.

## Methods

### Materials and method

All chemicals were purchased from Sigma Aldrich, VWR chemicals or Acros. They were used without purification unless mentioned. 2,2′-azobis(2-methylpropionitrile) (AIBN) was crystallized in hot methanol. Poly(N-vinyl-2-pyrolidinnone) (PVP) from Acros had a molecular weight of 3500 gmol^−1^. Polymers were made using the same method as described below, the details of which are shown in Table 1.

Before use, the glycidyl methacrylate (GMA) and monomer were passed through a plug of basic Al_2_O_3_. To a round-bottom 100 mL flask, 50 mL of tetrahydrofuran (THF), 5.8 g. (40.8 mmol) of GMA, and a co-monomer were added. The solution was stirred for 30 min and through which nitrogen was bubbled. Next, AIBN (radical initiator) was added. The flask was equipped with a condenser and heated at 65 °C for 12 h under nitrogen. The resultant solution was cooled down at 22 °C and poured into 10 volumes of diethyl ether. The supernatant was decanted, and the polymer was dried under vacuum at 60 °C for 12 h.

### Characterization

FTIR spectra were recorded on an Agilent Cary 630 equipped with an ATR accessory. Liquid ^1^H NMR analysis was performed on a Bruker 300 instrument operating at 300 MHz with an acquisition time of 1.9 s. Samples were dissolved in CDCl_3_ or DMSO d-6 (a TMSPA-based polymer) and the residual solvent peak was used for calibration. The solid-state ^13^C NMR spectrum was recorded on a Bruker Avance III HD operating at 100.60 MHz, using a triple-resonance 1.9 mm MAS probe in double resonance mode. The sample had a mass of 15 mg and the spinning frequency was 20 kHz. ^13^C spectra were obtained using 1.5 ms cross-polarization ramped from 70–100% of the maximum amplitude or a 90° pulse. The molecular weight distribution (Mn, Mw and PDI) was determined with a GPC (1260 Infinity II Multi-detector from Agilent) equipped with triple detection (LS at 690.0 nm, viscosity, and RI) and operating at 25 °C with THF as the eluent. The columns were Agilent (Varian) PL-Gel mixed-B 10 mm.

### Battery and cycling

The 2 Ah battery was composed of a stack of biface cathodes and anodes with polyethylene separators. The cell was 8 mm thick (energy: 92 Whkg^−1^, 171 WhL^−1^; power: 1923 Whkg^−1^, 3571 WhL^−1^).

Carbon-coated LiMn_0.75_Fe_0.20_Mg_0.05_PO_4_ (LMFP) was graciously supplied by Sumitomo Osaka Cement and carbon-coated Li_4_Ti_5_O_12_ (LTO) was made using the method published^30^. LiPF_6_, solvents and poly(vinylene difluoride) (PVDF) were purchased from BASF. Electronically conductive material such as Acetylene black and VGCF were purchased from Denka and Showa Denko, respectively.

Cathodes contained LMFP (90 wt%), acetylene black (4.0 wt%), and VGCF (1.0 wt%), and PVDF as a binder (5.0 wt%). The slurry mixed using a Thinky mixer, was coated on a 15 μm aluminum collector using the Doctor Blade method. The anodes contained LTO (90 wt%), acetylene black (5.0 wt%), and PVDF binder (5.0 wt%). The slurry, mixed using a Thinky mixer, was coated on a 15 μm aluminum collector using the Doctor Blade method. The poly(ethylene) separator was 16 μm thick. The electrolyte was LiPF_6_ 1.0 molkg^−1^ with carbonate solvents as reported in ref. ^30^.

Prior to extensive cycling, the cells were cycled at 0.2 C for 3 cycles at 25 °C. Subsequently, cycling was done at 1 C using a Biologic BCS-815 in a climate chamber operated at 45 °C. Cycling was stopped when the cells retained 80% of their initial capacity.

## Supplementary information

Supplementary Information.
