# Supplementary material for: Novel polymer coating for chemically absorbing CO2 for safe Li-ion battery
Source: Sci Rep. 2020 Jun 25;10:10305. doi: 10.1038/s41598-020-67123-1 (PMC7316716; doi:10.1038/s41598-020-67123-1)
Supplement: Supplementary file 1 — Supplementary Information. [file 41598_2020_67123_MOESM1_ESM.docx]

**Supplementary Information**

**Novel polymer coating for chemically absorbing CO_2_ for safe Li-ion battery**

Jean-Christophe Daigle, Yuichiro Asakawa, Martin Dontigny, Alexis Perea and Karim Zaghib

**Fig. S1.** ^1^H NMR spectrum of polymer 1

Incorporation of glycidyl methacrylate was calculated with this equation based on assignments reported[1, 2]:

x = mol GMA and y = mol MA

2x = 2.00 (Hd) ∴ x = 1

3y = 2.26 (He) ∴ y = 0.753

(x / (x +y)) x 100 = 57 mol% of GMA

**Fig. S2.** ^1^H NMR spectrum of polymer 2

Incorporation of glycidyl methacrylate was calculated with this equation based on assignments reported[1, 2]:

x = mol GMA and y = mol nBa

2x = 2.00 (Hd) ∴ x = 1

2y + x = 1.49 (He + Ha)

2y = 0.49 ∴ y = 0.245

(x / (x +y)) x 100 = 80 mol% of GMA

**Figure S3**. ^1^H NMR spectrum of pol(glycidyl methacrylate-co-3-(trimethoxysilyl)propyl methacrylate) (solvent: DMSO-d6)

Incorporation of glycidyl methacrylate was calculated with this equation based on assigments reported[2, 3]:

x = mol GMA and y = mol TMSPA

2x = 2.00 (Hd) ∴ x = 1

11y + x = 1.93 (Ha + He + 9H (Si-O-CH_3_))

11y = 1.93 – 1

y = 0.93/11 ∴ y = 0.085

(x / (x +y)) x 100 = 92 mol% of GMA

**Figure S4**. GPC trace of polymer 1

**Figure S5.** GPC trace of polymer 2

**Figure S6.** Solid-state ^13^C NMR spectrum of polymer 1 after carbon dioxide exposure.

$\left( \frac{w polymer}{Mw polymer} \right)x yGMA=y mol GMA in polymer$

$\frac{y mol GMA x \% conversion \left( FTIR \right)}{n polymer (mol)}=\frac{n CO2 (mol)}{n polymer (mol)}$ Equation S1

**Figure S7.** a) Charge-discharge curve of 2 Ah cell without trapping sheets. b) Cell with trapping sheets.

**Figure S8.** Cycle-life curve of 2 Ah cell with trapping sheets. (1C, 45^o^C)

**Figure S9**. Picture of pouch cell without trapping sheets during cycling (inflation occurred)

[1] K.M. Skupov, J. Hobbs, P. Marella, D. Conner, S. Golisz, B.L. Goodall, J.P. Claverie, Kinetic and Mechanistic Aspects of Ethylene and Acrylates Catalytic Copolymerization in Solution and in Emulsion, Macromolecules, 42 (2009) 6953-6963.

[2] J.-C. Daigle, Y. Asakawa, A. Vijh, P. Hovington, M. Armand, K. Zaghib, Exceptionally stable polymer electrolyte for a lithium battery based on cross-linking by a residue-free process, Journal of Power Sources, 332 (2016) 213-221.

[3] C. Feng, X. Pang, Y. He, Y. Chen, G. Zhang, Z. Lin, A versatile strategy for uniform hybrid nanoparticles and nanocapsules, Polymer Chemistry, 6 (2015) 5190-5197.
